# A Kirmes at Buffalo

**Published:** 1890-02-08

**Authors:** 


### Fallow Crops.

### A KIRMES AT BUFFALO.

Human nature, we know, is much the same all the world over, and we are not altogether surprised, therefore, that even the abilities of our American cousins have not as yet proved capable of arousing the average citizen to a keen interest in the affairs of the hospitals of his neighbourhood, or failing that to an automatic discharge of his duty in respect of them.

The General Hospital at Buffalo, New York, originated and established like so many of our own institutions, by the act of private citizens, and left like ours to be supported by so-called public charity, encounters the same financial difficulties, and must perforce adopt the same expedients for raising funds as our own unendowed hospitals have been practising for many a long year. But, just as genius is able to inspire colder natures with some of its own exquisite sensibilities, and to confer upon the procession of life's commonplaces the rank of a pageant, so the nervous energies of the American have succeeded in the attempt to invest hospital begging with novel and attractive features.

To us in England, bazaars, fairs, balls, carnivals, and so-called art unions are not unfamiliar. But for originality in design and elaboration of detail what have we had to compare with the Kirmes of Buffalo? The very title is attractive in the sense that it arrests attention. Naturally, everybody would ask what it meant, just as in all probability an English reader will ask now. Anticipating this question, the promoters have considerately printed an opening chapter in the programme under the title "What a Kirmes is." We learn that "the word translated literally means a church fair," and "to the untutored foreigner looks very much like a jolly rustic thanksgiving."

The particular Kirmes we are writing of took place at Music Hall, Buffalo, in aid of the General Hospital. Among the entertainments, dancing, it appears, occupied a prominent place, and while it is possible that even the good Bishop of Bedford might look askance at an announcement that "in the Swedish dance twelve pretty maidens trip along with their beaux in rollicking abandon," it is reassuring to hear from an independent source that the scene was one of "remarkable loveliness," and that the tableau drew "a mighty burst of applause from the audience." Perhaps it would have been more correct to have written "spectators," though it is only fair to make some allowance for a writer "filled with enthusiasm."

With so practical a people music and dancing were not likely to exhaust the programme. The Kirmes of Buffalo was also a "Kalender Fest," and each of the twelve booths, emblematic of the months, contained something to sell. It might not be altogether in harmony with our views to recommend the January dolls by saying that "though 'they neither toil nor spin,' yet we might be willing 'to be arrayed like one of these,'" and perhaps our insular appetites might rebel against a menu of "salads, oysters, ices, and coffee," but there can be no doubt of the energy infused into the undertaking, or of the general arrangements having been such as to command success. Every page of the dainty report before us is suggestive of real concern for the work undertaken, and unless appearances are unusually deceptive, of ungrudging co-operation and most remarkable self-subordination by a multitude of workers. Indeed, one wonders how the army of "chairmen"—mostly ladies, curiously enough—and committees could contrive to get on without friction. The absence of any supreme authority and the existence of a number of co-equal bodies all claiming power in their several departments, would appear to us fatal to perfection of organization, and we shudder to think of the unhappy condition to which the executive officers, who worked under eight separate committees, must have been reduced ere the Kirmes and Kalender Fest had completed its four days' carnival.

Can it be that committees in Buffalo are like some of our own, which make a brave show in print, but are poor things when it comes to work? Anyhow, while we heartily congratulate our friends at Buffalo upon the very original and inviting programme of their Kirmes, we cannot forbear sympathizing with them that despite their newer civilization, it is necessary to invoke the aid of dinners, balls, routs, salads, oysters, ices, and coffee, in order to render philanthropy equal to the discharge of one of the first and most obvious duties of an enlightened community.

Agricola.

